# Role of microRNA-34b-5p in cancer and injury: how does it work?

**DOI:** 10.1186/s12935-022-02797-3

**Published:** 2022-12-01

**Authors:** Xuechun Bai, Lianwen Zheng, Ying Xu, Yan Liang, Dandan Li

**Affiliations:** The Second Hospital of Jilin University, Changchun, Jilin China

**Keywords:** Cancer, Injury, microRNAs, miR-34b-5p, Pathogenesis

## Abstract

MicroRNAs (miRNAs or miRs) are a class of noncoding single-stranded RNAs that can regulate gene expression by binding to the untranslated sequences at the 3 ' end of messenger RNAs. The microRNA-34 family is dysregulated in various human diseases. It is considered as a tumor-suppressive microRNA because of its synergistic effect with the well-known tumor suppressor p53. As a member of the miRNA-34 family, miR-34b-5p serves as a powerful regulator of a suite of cellular activities, including cell growth, multiplication, development, differentiation, and apoptosis. It promotes or represses disease occurrence and progression by participating in some important signaling pathways. This review aimed to provide an overview and update on the differential expression and function of miR-34b-5p in pathophysiologic processes, especially cancer and injury. Additionally, miR-34b-5p‐mediated clinical trials have indicated promising consequences for the therapies of carcinomatosis and injury. With the application of the first tumor-targeted microRNA drug based on miR-34a mimics, it can be inferred that miR-34b-5p may become a crucial factor in the therapy of various diseases. However, further studies on miR-34b-5p should shed light on its involvement in disease pathogenesis and treatment options.

## Introduction

MicroRNAs (miRNAs or miRs), 21–24 nucleotides in length, are small, single-stranded noncoding RNAs that regulate gene expression at the post-transcriptional level through target mRNA cleavage or translational inhibition. The process of their generaton is usually divided into two steps: (i) genomic DNA genetic information transcription by RNA polymerase II to produce primary miRNA(pri-miRNA) transcript, which contains one or a few stem-loop structures consisting of approximately 70 nucleotides each; and (ii) processing of pri-miRNA by a microprocessor, Dicer-like 1 protein, into precursor miRNA (pre-miRNA), which is also a stem-loop structure and finally becomes mature miRNA by modification [1]. The mature miRNA is incorporated into an RNA-induced silencing complex. They recognize target mRNAs through imperfect base pairing and commonly result in the translational inhibition or destabilization of the target mRNA.

Disclosing the biological functionality of miRNAs is generally implemented by animal knockout models and transgenic overexpression experiments [2]. Functional studies indicate that miRNAs regulate practically every cellular process investigated so far, such as cell proliferation, differentiation, immune response, metastasis, senescence, autophagy and apoptosis, via regulating housekeeping genes and involving in various cell signaling pathways [3]. The changes in their expression are associated with many human pathologies [4–6]. The interesting thing is that the functions of miRNAs depend on different pathological types and physiological environments [3]. When miRNA is located in the cell plasma, it can act on the mRNA 3′-untranslated region (UTR) like a fire extinguisher, blocking the translation of mRNA and then exerting the negative regulation of genes. In contrast, when it is located in the nucleus, it serves as an igniter that changes the chromatin state of enhancers by binding to enhancers, thereby activating the transcriptional expression of genes.

The miR-34 family has been extensively studied and considered as tumor suppressor RNA because of its synergistic effect with the tumor suppressor p53 [7]. It is a tiny fragment located in the sub-band 1 of band 3 in the long arm 2 region of chromosome 11, including three members of miR-34a, miR-34b, and miR-34c. It is highly conserved during the evolutionary process. MiR-34 family acts as an antitumor agent by participating in some important signaling pathways or regulating multiple target mRNAs and proteins [8], such as phosphatidylinositol 3-kinase–protein kinase B signaling pathway (PI3K–Akt), Notch signaling pathway, cyclin dependent kinase (Cdk), and silent mating type information regulation 2 homolog 1 (SIRT1), promoting tumor cell apoptosis, inhibiting the proliferation and differentiation of tumor cells, hindering the invasion and migration of tumor cells, and enhancing immune surveillance [9]. In addition, recent studies have put forward that miR-34 family members not only assume the function of repressors in the development of tumors but also contribute to the pathogenesis of other diseases, such as regulating reproductive and nervous system function, influencing inflammatory and immune responses [10–13].

As a member of the miR-34 family, the altered expression patterns of miR-34b-5p play a key role in a variety of human diseases. The genetic inactivation of miR-34b-5p can influence the repression effects on its target gene, mRNA, or protein, particularly if the targets are functionally linked. If these problems are not controlled, changes in protein expression and cellular dysfunction often ensue, which may lead to disease [14–19]. For example, one study showed the deregulation of miR-34b-5p in patients with bladder carcinoma of aggressive phenotype compared with nonaggressive participants [20]. Another study indicated that miR-34b-5p inhibited aquaporin-2 to promote lipopolysaccharide-induced injury in human renal tubular epithelial cells [21]. LncRNA is one of the upstream regulators of miR-34b-5p, which inhibits the downstream target genes by binding to miR-34b-5p through sponge action, thereby regulating biological processes such as cell proliferation and apoptosis [22]. In contrast, the autoregulatory feedback on microprocessor expression is instrumental for balancing the efficiency and specificity of its activity by tuning effectively the microprocessor levels to those of its pri-miRNA substrates [23].

Although studies suggest that miR-34b-5p regulates various diseases, its pathogenic mechanisms primarily focus on tumor and cell injury. Thus, in this review, we focused on cancer and injury to overview and update the changes in the functional regulation, cellular communication, and pathogenesis of miR-34b-5p. Besides, findings on its mechanisms might provide guidance and novel ideas for detecting, diagnosing, and treating miR-34b-5p-related diseases.

## MiR-34b-5p in cancer

### Role of miR-34b-5p in colorectal cancer

**Fig. 1:**
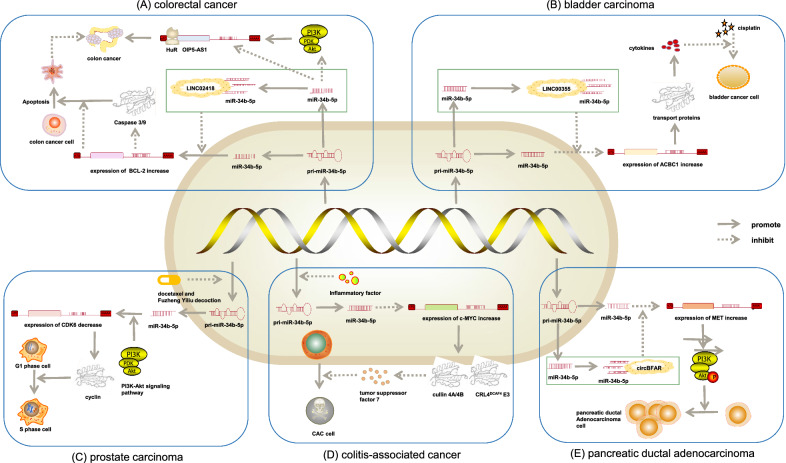
Mechanism of miR-34b-5p in the development of cancer. **A** Role of miR-34b-5p in colorectal cancer: LINC02418 promoted colon cancer progression by suppressing apoptosis via interaction with miR-34b-5p/BCL2 axis. HuR bound to OIP5-AS1 and stabilized OIP5AS1 expression while miR-34b-5p inhibited the proliferation and invasion of CC cells by inhibiting the OIP5-AS1 and PI3K/Akt pathways. **B** Role of miR-34b-5p in bladder carcinoma: Exosomal LINC00355 derived from cancer-associated fibroblasts promoted bladder cancer cell resistance to cisplatin by regulating miR-34b-5p/ABCB1 axis. **B** Role of miR-34b-5p in prostate carcinoma: The process of enhancement of anticancer activity of docetaxel by combination with Fuzheng Yiliu decoction in a mouse model of castration-resistant prostate cancer involved the regulation of miR-34b-5p and PI3K/Akt signaling pathways. **D** Role of miR-34b-5p in colitis-associated cancer: Inflammatory factors influenced the expression of c-MYC and CRL4DCAF4 E3 ligase activity by downregulating of miR-34b-5p to cause colitis-associated cancer. Role of miR-34b-5p in pancreatic ductal adenocarcinoma: Circular-RNA circBFAR promoted the progression of pancreatic ductal adenocarcinoma via the miR-34b-5p/MET/Akt axis

A study related to human colorectal cancer (CRC) proved that the expression of LINC02418 in CRC tissues was markedly higher than that in normal tissues; moreover, the patients with CRC having a high expressional level of LINC02418 had lower overall survival rates [24]. This long noncoding RNA contained a binding sequence for miR-34b-5p. Hence, it was deduced that LINC02418 might exert its biological effect via binding to miR-34b-5p [25]. The cell signaling pathway analysis revealed that LINC02418 adsorbed to miR-34b-5p via sponge action to hinder its binding to B-cell lymphoma-2 (Bcl-2), thus preventing the degradation of Bcl. It is well known that Bcl-2 inhibits the release of cytochrome c and pro-apoptotic factors so that the downstream caspase pathway is not able to activate, decreasing the expression of caspase 9 and caspase 3 [26]. Consequently, cell apoptosis is curbed, rendering cancer cells to growth, mobility, invasion, escape from cell death, and re-entry into abnormal cell cycles [27]. The study underlined that LINC02418 acted as a tumor driver by negatively regulating cell apoptosis through the miR-34b-5p/Bcl-2 axis, indicating that the LINC02418/miR-34b-5p/Bcl-2 axis was one potential indicator for prognosis prediction and a promising therapeutic target for CRC treatment (Fig. 1A) [16].

In another study, the combination of miR-34b-5p and RNA-binding protein human antigen R (HuR) could decrease the level of lncRNA opa-interacting protein 5 antisense transcript 1 (OIP5-AS1) by combining with OIP5-AS1 and prevent the PI3K–Akt pathway. Consequently, the tumor growth rate and tumor weight significantly decreased, finally impeding colon cancer (CC) progression (Fig. 1A) [28]. OIP5-AS1 is elevated in various cancers serving as a sponge against HuR [29], which has been found to promote proliferation, migration, invasion, and apoptosis of CC [30]. HuR is a highly abundant protein in cancers, targeting mRNAs to encode proteins that promote different aspects of tumorigenesis, such as cell proliferation, survival, invasion, and metastasis [29].

### Role of miR-34b-5p in bladder carcinoma

MiR-34b-5p overexpression or ABCB1 silencing enhanced the sensitivity of bladder carcinoma (BC) cells to cisplatin by inhibiting cell viability and facilitating cell apoptosis in cisplatin-exposed BC cells [31]. LINC00355 acts as a competing endogenous RNA by sponging miR-34b-5p, whose target mRNA is ABCB1, to prevent it from binding to ABCB1 mRNA and upregulate ABCB1 expression; this causes BC cell proliferation as well as the inhibition of BC cell apoptosis (Fig. 1B) [32]. ABCB1 inhibits the uptake of orally administered drugs and contributes to multidrug resistance in cancer cells via exporting various exogenous compounds [33]. A study aimed to determine candidate miRNAs as prognostic biomarkers for differentiating the aggressive type of BC, and verified the deregulation of miR-34b-5p in patients with BC of aggressive phenotype compared with nonaggressive participants to promote the proliferation of cancers [20]. As a consequence, miR-34b-5p may be used not only as a diagnostic marker to identify patients with BC, but also for treating BC via mimics transfection.

### Role of miR-34b-5p in prostate carcinoma

The analysis of putative target genes of castration-resistant prostate cancer indicated that specific downregulation of miR-34b-5p after the combination therapy of docetaxel and Fuzheng Yiliu decoction might hinder the PI3K–Akt signaling pathway to suppress cell survival, growth, and proliferation, and facilitate apoptosis resulting in enhanced anticancer effects (Fig. 1C) [34]. Therefore, limiting the expression of miR-34b-5p and silencing PI3K–Akt could restrain the development of castration-resistant prostate cancer and present a promising therapeutic strategy for this cancer.

### Role of miR-34b-5p in colitis-associated cancer

The findings of colitis-associated cancer (CAC) tumorigenesis demonstrated that the expression of miR-34b-5p was constrained due to intracellular inflammation and DNA hypermethylation, which restricted its inhibitory effect on transcription factor proto-oncogene proteins c-myc (c-Myc). They activated the downstream events, including the activation of cullin 4A and 4B (CUL4A/4B) and the induction of CRL4^DCAF4^E3 ligase activity. CRL4^DCAF4^E3 ligase ubiquitinated the suppression of tumorigenicity 7 and led to its degradation, eventually resulting in CAC tumorigenesis (Fig. 1D) [7, 35].

### Role of miR-34b-5p in pancreatic ductal adenocarcinoma

An analysis of a cohort of 208 patients with pancreatic ductal adenocarcinoma (PDAC) showed that the expression of miR-34b-5p in PDAC tissues declined and was negatively associated with the tumor-node-metastasis stage. Besides, circular-RNA BFAR (circBFAR), which was highly expressed in PDAC, upregulated the expression of the mesenchymal–epithelial transition (MET) factor via sponging miR-34b-5p. It also activated the downstream phosphorylation of Akt and further activated the MET/PI3K/Akt signaling pathway to ensure the multiplication and differentiation of PDAC cells, which ultimately promoted the progression of PDAC (Fig. 1E) [17]. In brief, this consequence provided evidence to support that the binding of circBFAR and miR-34b-5p might be a promising site for clinical MET-targeted therapy in PDAC.

### Role of miR-34b-5p in lung cancer

**Fig. 2:**
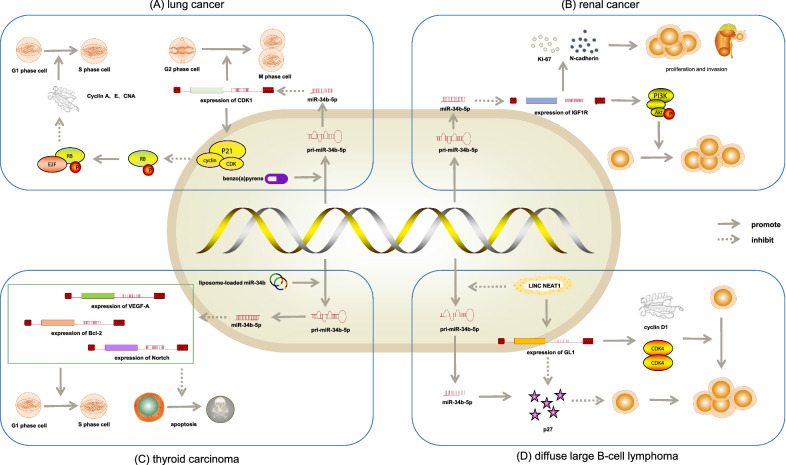
Mechanism of miR-34b-5p in the development of cancer. **A** Role of miR-34b-5p in lung cancer: High levels of miR-34b-5p in the lungs inhibited Cdkn1 of p53, Then the appearance of p21 protein, cyclin and CDK complex caused the decrease in RB protein phosphorylation, thereby inhibiting the expression of E2F and cyclin A and E; CNA downregulation led to the cell cycle arrest. **B** Role of miR-34b-5p in renal cancer: The expression of miR-34b-5p was upregulated, which decreased the level of IGF1R. Also, the PI3K/Akt signal transduction pathway was inhibited, thereby reducing the proliferation, invasion, and metastasis of renal cancer cells. **C** Role of miR-34b-5p in thyroid carcinoma: MiR-34b-5p could influence angiogenesis in thyroid carcinoma by changing endothelial cell proliferation through reduced VEGF-A level, arrest cell cycle and induce apoptosis. **D** Role of miR-34b-5p in diffuse large B-cell lymphoma: Nuclear paraspeckle assembly transcript (NEAT)1 inhibited cell proliferation and promoted cell apoptosis through decreasing miR‑34b‑5p level and upregulating *GLI1*

Since benzo(a)pyrene (BaP) exposure results in lung cancer, several studies discovered prominent upregulation of miR-34b-5p in mice exposed to BaP. The consequences also revealed that high levels of miR-34b-5p in the lungs might cause changes in the expression of critical downstream target cyclin-dependent kinase inhibitor (Cdkn)1 of p53 to repair the damage caused by BaP in the lungs. Further, it was concluded that the activation of miR-34b-5p was related to the change in the cell cycle because Cdkn1 was an indispensable factor in controlling the cell cycle (Fig. 2A) [36, 37]. It was realized that miR-34b/c methylation was a collective alteration in small-cell lung cancer (SCLC). As a consequence, decreased normal miR-34b/c expression might confer tumor cell proliferation and invasiveness [38]. Another study showed that the aberrant DNA methylation of miR-34b/c was associated with a high probability of recurrence (*P* = 0.026) and correlated with low overall survival (*P* = 0.010) and disease-free survival (*P* = 0.017) in lung cancer [39, 40]. These results strongly indicated that miR-34b/c was part of the pathogenesis of SCLC and presumably played a vital role as the therapeutic target in SCLC. Nevertheless, the detailed mechanism underlying the role of miR-34b-5p in SCLC is not clear. Hence, further studies are required to identify whether these molecular signatures can be used as markers for screening or another biomarker of immunotoxicity to diagnose lung cancer and forecast the prognosis.

### Role of miR-34b-5p in renal cancer

The results of an experiment displayed that the upregulation of miR-34b-5p significantly reduced the proliferation and invasion of renal cancer cells, suggesting that it might play a vital role as a tumor suppressor gene in renal cancer. The potential target gene of miR-34b-5p is the type 1 insulin-like growth factor receptor (IGF1R), whose abnormal expression is closely related to the occurrence and development of a variety of malignant tumors [41]. When the expression of miR-34b-5p was upregulated in renal carcinoma cells, the expression of the IGF1R gene significantly decreased. The PI3K/Akt signal transduction pathway was inhibited after IGF1R gene decreased. Further, the levels of PI3K, p-Akt and p-extracellular regulated protein kinases, as the PI3K/Akt signal transduction pathway proteins, decreased, thereby reducing the proliferation, invasion, and metastasis of cells. At the same time, the levels of Ki-67 and N-cadherin act, as proliferation-related and invasion-related proteins, reduced suggesting that renal cancer cells had reduced proliferation and invasion (Fig. 2B) [42].

Through gathering preoperative urine samples from patients who had histologically certified clear cell renal cell carcinoma(ccRCC), it was found that miR-126-3p combined with miR-34b-5p could prominently distinguish patients with ccRCC from healthy participants and was also able to recognize small renal masses (pT1a, ≤ 4 cm) more sensitively[43, 44]. Therefore, we believed that miR-34b-5p might become a predictor and treatment direction of renal malignant renal tumors.

### Role of miR-34b-5p in thyroid carcinoma

The expression of miR-34b was low in anaplastic thyroid carcinoma cells. However, its apparent overexpression was perceived with transfected liposome-loaded miR-34b in thyroid carcinoma cells and mouse xenografts. Also, the diminution in VEGF-A, Bcl-2, and Notch1 protein translation, declined cell proliferation, retarded wound healing, impeded cell cycle progression, and aggrandized apoptosis occurred. Further, the expression of miR-34b were obviously related to T-stages of thyroid carcinomas (*P* = 0.042) [45]. Some experimental results suggested that miR-34b-5p could influence angiogenesis in thyroid carcinoma by changing endothelial cell proliferation through reduced VEGF-A secretion in the extracellular matrix. Also, miR-34b-5p caused cell cycle arrest via accumulation of cells in the G0–G1 phase, blocked their entry into the S transitional phase, and induced apoptosis in anaplastic thyroid carcinoma cells (Fig. 2C) [46, 47]. These results indicated that miR-34b-5p inhibition might play an indispensable role in the proliferation and metastasis of thyroid carcinoma. Multiple targeted agents are currently available for treating malignancies [48, 49]. The surveys verified the tumor suppressor properties of the miR-34b family by VEGF-A regulation in thyroid carcinoma. It is expected that the delivery of miR-34b-5p using cationic liposomes may be a useful, targeted therapeutic strategy in thyroid carcinoma.

### Role of miR-34b-5p in diffuse large B-cell lymphoma

In an experiment, Qian et al. found that nuclear paraspeckle assembly transcript *(NEAT)1* and GLI family zinc finger (GLI*)*1 were upregulated while miR‑34b‑5p was downregulated in diffuse large B-cell lymphoma (DLBCL) tissues and cell lines compared with the control group. The other finding was that the knockdown of *NEAT1* or the overexpression of miR‑34b‑5p inhibited cell proliferation and promoted cell apoptosis [50]. Moreover, the expression of proliferation-related proteins, such as GLI1, cyclin D1, and CDK4 [51], decreased accompanied by increased p27 expression after miR-34b-5p overexpression. It was inferred that *NEAT1* acted as a competing endogenous RNA, regulating the miR‑34b‑5p–GLI1 axis through declining the miR‑34b‑5p level and increasing *GLI1* level, further accelerating the proliferation of DLBCL cells, which was often associated with disease progression and poor prognosis (Fig. 2D) [52]. In other words, the NEAT1–miR‑34b‑5p–GLI1 axis played an important role in the progression of DLBCL and could provide a novel therapeutic target for DLBCL.

### Role of miR-34b-5p in leukosis

**Fig. 3:**
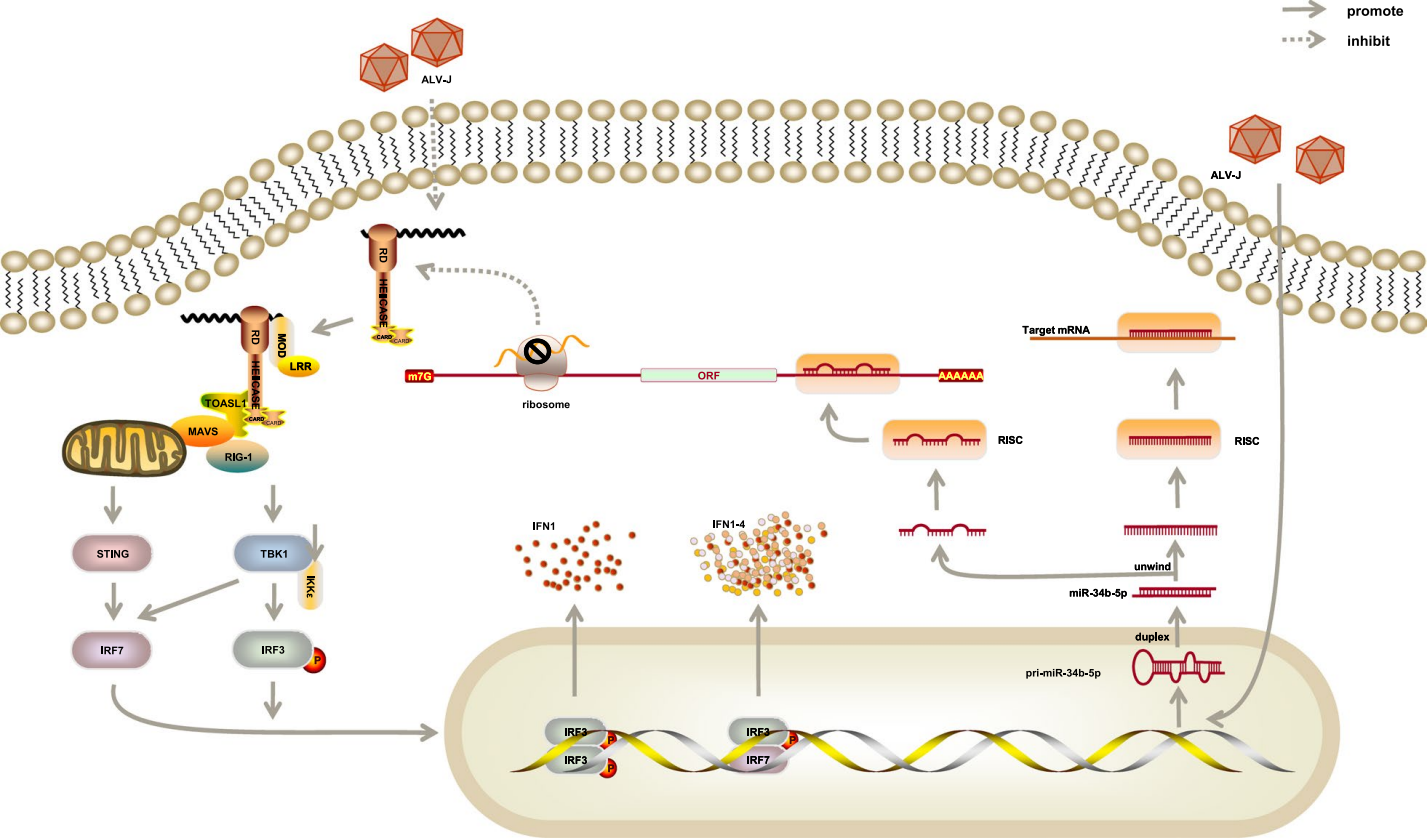
Signaling pathway activated by miR-34b-5p after Avian Leukosis Virus Subgroup J infection

Avian Leukosis Virus Subgroup J (ALV-J) can induce myeloid leukosis, various tumors, growth retardation, and serious immunosuppression [53]. Previous data showed that miR-34b-5p was significantly upregulated to accelerate the proliferation of ALV-J-infected cells by inducing the progression from the G2 phase to the S phase and promote cell migration in ALV-J-infected chicken spleens. However, melanoma differentiation − associated gene 5 (*MDA5*) had the opposite expression pattern. The ectopic expression of *MDA5* inhibited cell proliferation, cell cycle, and cell migration in ALV-J infection. A previous study proved that the *MDA5* signaling pathway inhibited the mRNA and protein expression of the ALV-J env gene and suppressed virion secretion by activating the interferon (IFN) signaling pathway (Fig. 3) [54]. On the contrary, the abnormal expression of miR-34b-5p suppressed the function of *MDA5*, which triggered the signal transduction cascade to induce an IFN-β response and, in turn, upregulated downstream antiviral genes, such as interferon promoter stimulator 1 *(IPS-1), IFN-β,* oligoadenylate synthetase *(OAS),* myxovirus resistance gene *(Mx)-1,* and major histocompatibility complex *(MHC)* class I. MiR-34b-5p inhibited the expression of *MDA5*, led to increased proliferation and migration of the ALV-J-infected cells, and promoted ALV-J replication [55].

The expression of miR-34b-5p was significantly upregulated in ALV-J-infected chicken spleens while melanoma differentiation-associated gene 5 had the opposite expression pattern. It could activate the interferon signaling pathway and the MDA5 signaling pathway to inhibit the mRNA and protein expression of the ALV-J env gene and suppress virion secretion. In addition, miR-34b-5p significantly inhibited the expression of genes in the MDA5 signaling pathway, including MDA5, IPS-1, IFNβ, OAS, Mx1, and MHC class I, to accelerate the proliferation and migration of ALV-J-infected cells.

## MiR-34b-5p in injury

### Role of miR-34b-5p in respiratory system injury

MiR-34b-5p is predominantly expressed in the lung tissue [36]. The level of miR-34b-5p increased 7.3-fold in acute lung injury (ALI), which was the time point where the progranulin (PGRN) expression was greatly diminished. It was inferred that the upregulated miR-34b-5p might play a significant role by downregulating the expression of PGRN in ALI. The universal comprehension of PGRN manifested that it could increase survival by decreasing lung inflammation and relieving apoptosis [56]. Another study found that by declining the PGRN level, the augmentation of miR-34b-5p could recruit inflammatory cells, produce inflammatory stroma, regulate epithelial cell apoptosis, and damage lung tissue [57]. Taurine upregulated 1 (TUG1) is an RNA gene, which interacts with the polycomb repressor complex and is involved in the epigenetic regulation of transcription, promoting cell proliferation. A previous study showed that TUG1 was involved in the inflammatory response of ALI via declining the expression of the downstream target named miR-34b-5p [58]. Additionally, lncRNA TUG1 protected alveolar epithelial cells against inflammation by attenuating the transcriptional activity of miR-34b-5p and increasing the expression of Grb2-associated binding protein indirectly [59]. These findings indicated that miR-34b-5p regulated by TUG1 could play an important role in treating sepsis-induced ALI.

The results of a study revealed that the ratio of wet to dry lung tissue weight decreased, and the apoptosis rate was also significantly lower in rats with acute respiratory distress syndrome (ARDS) after the upregulation of miR-34b-5p. The results also showed that miR-34b-5p decreased interleukin (IL)-6 and tumor necrosis factor (TNF)-α levels in the peripheral blood of rats with ARDS. As early inflammatory factors, IL-6 and TNF-α may reflect the severity of the inflammation and induce inflammatory chemokine production in the lung epithelium, which promotes inflammatory cell infiltration, increases cell permeability, exacerbates pulmonary edema, and ultimately leads to lung consolidation. These findings suggested that miR-34b-5p attenuated inflammatory cell infiltration and lung injury of rats with ARDS, reduced pulmonary edema, and inhibited the apoptosis of alveolar epithelial cells [60].

The results of a trial suggested that miR-34b-5p levels decreased significantly in human bronchial epithelial cells after respiratory syncytial virus infection [61]. Also, these changes mediated the induction regulation of the mucin expression gene MUC5AC through the activation of the c-Jun signaling pathway, DNA methylation, and histone modifications resulting in mucus deposition and airway obstruction, hence leading to further infection and even increasing bacterial colonization [62, 63]. A study suggested that miR-34b-5p could be a useful biomarker for influenza B detection [64]. The discovery of miR-34b-5p and its unique expression profile in patients with influenza offered a new way for the early diagnosis of influenza. It is expected that a noninvasive approach using the throat swab miRNAs may be an effective way for influenza diagnosis in the future.

An investigation suggested that miR-34b-5p played a versatile role in developing inflammation in bleomycin-induced fibrotic lung tissue in mice. To be specific, miR-34b-5p restricted extracellular matrix (ECM) degradation and enlarged the alveolar spaces continually via inhibiting the expression of tissue inhibitor of metalloproteinases-3 (TIMP3), which has been acknowledged as a pivotal regulator in lung homeostasis, to inhibit the progression of pulmonary fibrosis [65, 66]. TIMP3 was recognized as a direct target of miR-34b-5p in numerous studies since enhanced expression of miR-34b-5p led to a decline in TIMP3 expression and its knockdown was responsible for TIMP3 elevation. The research suggested that miR-34b-5p boosted bleomycin resistance by decreasing the expression of TIMP3 and further facilitated the fibrotic course of lung tissue [67].

A previous study showed a connection between idiopathic pulmonary arterial hypertension (IPAH) and and miR-34b-5p, which was associated with most of the declinable target differentially expressed genes, cell multiplication, and adhesion-independent growth [68]. The target genes of miR-34b-5p are basically related to immune and inflammatory reactions, for instance, neutrophil chemotaxis and migration, integrin binding, and Toll-like receptor binding [69, 70]. Besides, they are also involved in inflammation pathways such as the IL-17 signaling pathway, indicating that miR-34b-5p might play crucial roles in the pathogenesis of IPAH through enhanced inflammatory response [71].

### Role of miR-34b-5p in reproductive system injury

MiR‐34b‐5p might be involved in alternative splicing of the KIT proto-oncogene (kit)-l pre‐mRNA in murine ovarian granulosa cells through retrovirus-associated DNA sequences (Ras) signaling pathway, Rap1 GTP-binding protein (Rap)1 signaling pathway, FOXO protein (Foxo) signaling pathway, Hippo signaling pathway, mitogen-activated protein kinases (MAPK) signaling pathway, and carcinogenic pathway to affect biological processes of cell metabolism regulation, post-transcriptional regulation of mRNA, interleukin-6-mediated signal transduction, cell cycle, cell proliferation, differentiation, and migration [72]. The downstream target genes and proteins of these pathways are closely involved in abnormal cellular processes, such as cell proliferation, growth and differentiation, apoptosis regulation, oxidative stress adaptation, and inflammatory response, which are manifested as cytotoxic effects that cause ovarian granulosa cell injury and ovarian dysfunction [73]. Besides the aforementioned cellular pathways, a large number of genes, signal channels, and proteins affected by miR-34b-5p have been found in multifarious reproductive system diseases, which are the basic macromolecules of biology, regulating practically all cellular activities and functions (Table 1).

**Table 1:** Role of miR-34b-5p in multifarious reproductive system diseases

Diseases	Gene, mRNA, signal channels, or proteins that may be involved		Variation of miR-34b-5p	Influencing mechanism, effect, or role of miR-34b-5p	References
Ovarian function damage	Ras signaling pathway Rap1 signaling pathway Foxo signaling pathway Hippo signaling pathway MAPK signaling pathway Carcinogenic pathway		Down	Cell metabolism regulation post‐transcriptional Regulation of mRNA interleukin‐6‐mediated Signal transduction cell cycle Cell proliferation Differentiation and migration	Wang, W., et al., [72]
Ovarian cancer	Met gene c-met gene Bcl-2 gene p53 gene Myc gene Cdk6 gene	MET protein BCL-2 protein CDK4 protein	Down	Regulation of cell death Occurrence of epithelial ovarian cancer Development of epithelial ovarian cancer Metastatic clinical stage Proliferation and invasion Cell Proliferation Cell adhesion-independent growth	
Spermatogenesis	c-Kit gene cdk6 gene Rbm44 gene Cdh3 gene	CDK6 protein C-KIT protein	Up in spermatogenesis Down in decreases in spermatocytes	Cell developmental processes mRNA transcription and regulation Cell cycle regulation Signal transduction Protein modification Semen concentration and motility regulation	Sree, S., et al., [76] Smorag, L., et al., [77] Eikmans, M., et al., [79]
Placental function	BCL2 gene TP53 gene MYC gene CDKN1B/C gene VEGFA gene TNFSF10 gene ZEB1 gene		Up	Trophoblast proliferation and apoptosis Abnormalities in nutrient transport Endocrine function in adolescent damage Tissue remodeling Angiogenesis Placental development	Baker, B.C., et al., [74]
Endometrial endometrioid carcinoma	ESRRB mRNA SP7 mRNA GABRB2 mRNA B4GALNT mRNA		Down	Predict lymph node metastasis in endometrial Endometrioid carcinoma	
Minimal deviation adenocarcinoma of uterine cervix	Notch1 gene Notch2 gene miR-34b-5p/Notch1 pathway		Down	Promote development of cervical cancer Diagnostic biomarkers	Lee, H., et al., [55]

A large number of genes, signaling pathways, and proteins affected by miR-34b-5p have been found in multifarious reproductive system diseases, which are the basic macromolecules of biology, regulating practically all cellular activities and functions

A prospective study found that miR-34b-5p was upregulated during the low folate status, and the levels of Bcl-2, myc, vascular endothelial growth factor-A (VEGF-A), and zinc finger E‐box-binding homeobox 1 (ZEB1) were reduced as predicted by bioinformatics analysis. These factors invariably caused a high incidence of small-for-gestational-age infants, placental dysfunction, trophoblast apoptosis, amino acid transport reduction, and altered placental hormones [74, 75]. Therefore, it was speculated that miR-34b-5p was an underlying factor in regulating the expression of Bcl-2, myc, VEGF-A, and ZEB1, which maintained placental function.

Many studies investigated the effect of miR-34b-5p on spermatogenesis. MiR-34b-5p showed a progressive increase from prepubertal through pubertal to adolescent [76]. MiRNA signature of spermatogonial stem cell (SSC), and premeiotic (PrM) and meiotic cells revealed that specific miRNAs of SSC (miR-221), PrM (miR-203), and meiotic (miR-34b-5p) and their targets, c-Kit, Rbm44, and Cdk6, showed evidence for their functional relevance during spermatogenesis [77]. In certain observational studies, the levels of miR-34b-5p acquired from the seminal plasma in the control group were significantly higher than those in the asthenozoospermia and oligozoospermia groups. In addition, the miR-34b-5p level of testicular biopsies from patients with nonobstructive azoospermia was downregulated. These results confirmed that the downregulation of miR-34b-5p resulted in decreased sperm motility and quantity [78, 79]. The testicular miR-34b-5p level was downregulated in the ethylene glycol monomethyl ether (EGME)-induced testicular toxicity model in cynomolgus monkeys, suggesting that these spermatogenic cells were damaged by EGME treatment [80]. In the testicular–hyperthermia (TH) treatment–induced testicular injury model characterized by decreased numbers of spermatocytes and spermatids, the level of miR-34b-5p located in meiotic cells was decreased [81]. All these studies showed that miR-34b-5p was a crucial factor in spermatogenesis.

### Role of miR-34b-5p in nervous system injury

**Fig. 4:**
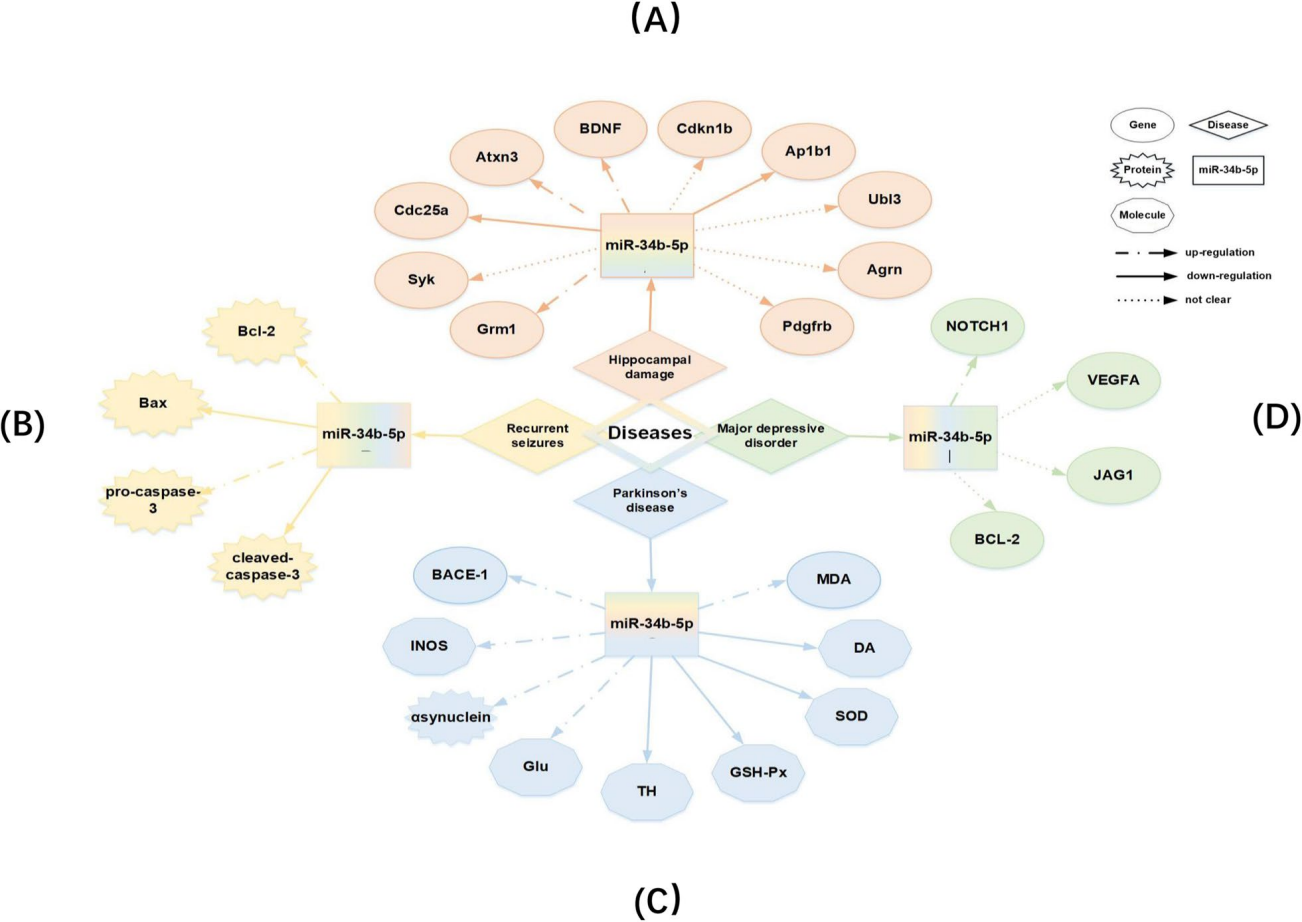
Relationship of neurological diseases, genes and miR-34b-5p. **A** The target genes of miR-34b-5p in hippocampal damage. *Grm1* Glutamate receptor, metabotropic 1. *Syk* Spleen tyrosine kinase. *Cdc25a* Cell division cycle 25 homolog A. *Atxn3* Ataxin 3. *BDNF* Brain-derived neurotrophic factor. *Cdkn1b* Cyclin-dependent kinase inhibitor 1**B**. *Ap1b1* Adaptor-related protein complex 1,beta 1 subunit. *Ubl3* Ubiquitin-like 3. *Agrn* Agrin. *Pdgfrb* Platelet-derived growth factor receptor,beta polypeptide. *B* The target genes of miR-34b-5p in recurrent seizures. **C** The target genes of miR-34b-5p in Parkinnson’s disease. *BACE1* β-Site amyloid precursor protein cleaving enzyme 1. *iNOS* inducible nitric oxide synthase. *Glu* glutamic acid. *TH* tyrosine hydroxylase. *GSH-Px* glutathione peroxidase. *SOD* superoxide dismuta. *DA* dopamine. *MDA* malondialdehyde. **D** The target genes of miR-34b-5p in major depressive disorder. *VEGF-A* vascular endothelial growth factor

While testing the expression of miRNAs in the offspring rat hippocampus after exposure to fluorine combined with aluminum (FA), it was found that miR-34b-5p was higher in the exposed group. At the same time, the protein levels of brain-derived neurotrophic factor (BDNF) and its receptor, tyrosine receptor kinase B (TrkB), were markedly downregulated in the hippocampus [82]. The combination of BDNF with TrkB causes the phosphorylation of TrkB and activation of intracellular signaling pathways, thus increasing the synthesis and release of neurotransmitters conducive to learning and memory [83]. Hence, researchers believed that miR-34b-5p might mediate FA-induced developmental neurotoxicity by downregulating the protein levels of BDNF and TrkB, thus participating in the mechanisms of hippocampal damage, which is key to the offspring's learning and memory damage caused by FA exposure during the embryonic stage and into adulthood [84]. In addition, numerous genes involved in the regulation of miR-34b-5p, such as Grm1, Syk, and BDNF, were closely related to learning and memory abilities (Fig. 4A).

A study based on a rat model of recurrent seizures induced by flurothyl treatments showed that miR-34b-5p expression was boosted significantly in the experimental group [85]. More surprisingly, this upregulation occurred simultaneously with astrocyte apoptosis, implying the involvement of miR-34b-5p in seizures by causing astrocyte apoptosis [86]. Further, miR-34b-5p targeted Bcl-2 mRNA directly and caused a reduction of Bcl-2 and pro-caspase-3 in astrocytes but an accumulation of Bax and cleaved caspase-3. This finding indicated that miR-34b-5p exerted a pro-apoptotic function by increasing Bax/Bcl2 ratio [85]. Additionally, miR-34b-5p modulated astrocyte apoptosis in response to kainic acid (KA) glutamate receptors, causing ROS production, and affecting mitochondria function, and induce cell death in astrocytes [87]. These data indicated that miR-34b-5p was involved in modulating neuronal injury in the early stage of convulsion-induced damage and played an important role in seizure (Fig. 4B) [88, 89].Therefore, considering that preventing neuronal death in early disease stages would greatly improve therapeutic outcomes, the therapies targeting miR-34b-5p might serve as a novel therapeutic way of curing neurological diseases.

A study using rat models of Parkinson’s disease (PD) established through the injection of 6-hydroxydopamine revealed that the downregulation of lncRNA BACE1-AS inhibited inducible nitric oxide synthase, α-synuclein, and glutamic acid activation and elevated dopamine and TH levels to improve oxidative stress injury in rats with PD by upregulating miR-34b-5p via integrating with it and indirectly downregulating BACE1 [90]. This restricted apoptosis and improved oxidative stress injury in the substantia nigra neurons of rats with PD. It was observed that the substantia nigra neurons contracted into a round shape, and the intracellular substances were centralized obviously when BACE1 expression increased [90]. It indicated that the suppression of BACE1 activity could negatively work on AD progression by controlling miR-34b-5p production. This was because it was a direct target gene of miR-34b-5p [91]. A previous study also demonstrated that the repression of miR-34b and miR-34c improved α-synuclein expression in PD, which further promoted PD pathogenesis (Fig. 4C) [92].

The expression levels of miR-34b-5p in patients with major depressive disorder (MDD) were significantly higher than those in control participants, while they were significantly lower in patients with suicidal tendencies. Nevertheless, its mechanism is not clear to date. It might be related to the regulation of the Notch1 gene and cognitive function because the levels of the Notch1 gene were significantly lower in patients with MDD at the same point when the levels of miR-34b-5p increased [93]. The Notch signaling pathway induced proliferation, differentiation of neural stem cells, and growth of nerve cell axons and dendrites to affect neuronal plasticity and be involved in MDD [94, 95]. Therefore, it was presumed that miR-34b-5p might promote MDD by inhibiting the Notch signaling pathway. In a global analysis relative to the healthy controls, the miR-34b-5p level prominently declined in depressed patients. Also, its targets, such as DNA-methyltransferase 3 beta, Bcl2, and vascular endothelial growth factor-A (VEGF-A), changed, all of which were previously implicated in depression [96]. Further, multiple studies proved that the differential expression of miR-34b-5p aggravated brain injury leading to depression-like phenotypic manifestations as it was associated with neuronal regeneration and astrocytic death (Fig. 4D) [85, 90].

### Role of miR-34b-5p in renal injury

A recent study showed that miR-34b-5p upregulation could suppress aquaporin-2 (AQP2) expression by binding to the 3′-UTR of AQP2, and increase the expression of pro-apoptotic proteins and proinflammatory cytokines to aggravate apoptosis and inflammation in LPS-induced renal tubular epithelial cells (HK-2) [21, 97]. Moreover, it was also found that AQP2 silencing abolished the negative effects of miR-34b-5p suppression on LPS-induced apoptosis and inflammatory response in HK-2 cells [98]. Both of them uncovered the mechanism of miR-34b-5p-mediated AQP2 in sepsis-induced injury, which is the crucial mechanism in renal injury.

### Role of miR-34b-5p in skin injury

Recently, a study showed that the transfection of miR-34b-5p mimics decreased the expression of both *collagen type I alpha 1 chain* and elastin but abundantly increased *matrix metallopeptidase (MMP)-1* expression. These factors were mainly involved in events such as cell adhesion, collagen synthesis, positive regulation of transcription and gene metabolism, and metabolism of collagen, synapse, and cytoplasmic vesicles [99], as well as insulin signaling pathway, erythroblastic leukemia viral oncogene homolog signaling pathway, and focal adhesion pathway. In addition, miR-34b-5p mimics in human dermal fibroblasts significantly induced cell cycle arrest, leading to abnormal cellular morphology, thus indicating negative impacts on the molecular markers for skin aging [100]. So far, the signaling pathway mostly investigated involves the interaction of these mimics with the target genes, including SIRT1, *c-Myc, c-Met*, and E2F3, which were regarded as genes for pro-longevity and cell cycle progression [101–103]. Therefore, considering the importance of miR-34b-5p in skin aging, further studies on the role of miR-34b-5p in skin aging should be conducted, providing possible biomarkers for skin aging research as well as potential targets for anti-aging therapies.

### Role of miR-34b-5p in vascular injury

The most key modules associated with vascular aging are triglyceride and free fatty acid–related genes, which are considered significant determinants of age-related vascular dysfunction. Interrelated research demonstrated that the hub genes for *Enpp5, Fez1, Kif1a,* and F3 with their interacting miRNAs, including miR-34b-5p, miR-449a, and miR-449c, exhibited the maximum connectivity with external lipid-related traits [104, 105]. Thus, their interactions may occur in age-related vascular dysfunctions, and hence they might work as potential biomarkers.

## Conclusion and future perspectives

On the one hand, miR-34b-5p could be downregulated by sponge adsorption, which relieved the inhibitory effect on downstream binding targets and promoted the proliferation, differentiation and invasion of tumor cells. On the other hand, when upregulating miR-34b-5p, tumor development was delayed by downregulating cell cycle-related proteins and increasing the expression of antitumor genes. An interesting finding is that via generating protective factors, participating in signaling pathways, or regulating gene expression, miR-34b-5p seems to act as a suppressor in cancers but as a stimulator in injury. However, there are few reports on the role its mechanism of miR-34b-5p in other systemic injuries. This may be because the function of miR-34b-5p has not yet been fully mined so that its role in other injuries has not received sufficient attention.

In this review, we provided an overview and update on different biological aspects and individual functions of miR-34b-5p. Most of the current studies on miR-34b-5p focus on the detection of expression levels and preliminary exploration of pathogenic mechanisms, while no standardized detection methods have yet been developed. In addition, the upstream and downstream regulatory network of miR-34b-5p remains unclear. It is speculated that the transfection of tumor cells with miR-34b-5p mimics to inhibit their proliferation and invasion and to repair cell damage using miR-34b-5p inhibitors may be new directions for future exploration, and the findings may translate into effective regimens for the treatment of tumors and injuries. This study was novel in summarizing the role of miR-34b-5p in the pathogenesis of a variety of cancer and injury, and mapping the miR-34b-5p related mechanistic pathways in graphical form so that the relevant research results were more clearly displayed. However, the manuscript fails to elucidate the specific protocol of miR-34b-5p in disease therapy because we need further studies to identify upstream and downstream mRNA signals associated with cancer as well as the background environment required for their interaction.

## Data Availability

All data generated or analyzed during this study are included in this published article.

## Abbreviations

miRNAs or miRs: MicroRNAs
nt: Nucleotides
pri-miRNA: Primary miRNA
pre-miRNA: Precursor miRNA
PDAC: Pancreatic ductal adenocarcinoma
TNM: Tumor-node-metastasis
circBFAR: Circular-RNA BFAR
MET: Mesenchymal–epithelial transition factor
OSCC: Oral squamous cell carcinoma
CAC: Colitis-associated cancer
CRC: Colorectal cancer
BCL2: B-cell lymphoma-2
ALI: Acute lung injury
PGRN: Progranulin
LPS: Lipopolysaccharide
ECM: Extracellular matrix
TIMP3: Tissue inhibitor of metalloproteinases-3
BaP: Benzo(a)pyrene
SCLC: Small-cell lung cancer
BC: Bladder carcinoma
AQP2: Aquaporin-2
HK-2: Human renal tubular epithelial cells
SSC: Spermatogonial stem cell
PrM: Premeiotic
EGME: Ethylene glycol monomethyl ether
TH: Testicular–hyperthermia
VEGF-A: Vascular endothelial growth factor-A
ZEB1: Zinc finger E-box-binding homeobox 1
EEC: Endometrial endometrioid carcinoma
LNM: Lymph node metastasis
MDA: Minimal deviation adenocarcinoma
DEMs: Differentially expressed miRNAs
CORT: Corticosterone
DLBCL: Diffuse large B-cell lymphoma
ALV-J: Avian leukosis virus subgroup J
MDA5: Melanoma differentiation-associated gene 5
IFN: Interferon
FA: Fluorine combined with aluminum
BDNF: Brain-derived neurotrophic factor
PD: Parkinson’s disease
SI: Social isolation
MDD: Major depressive disorder
IPAH: Idiopathic pulmonary arterial hypertension
DEGs: Differentially expressed genes

## References

[1] Achkar NP, Cambiagno DA, Manavella PA (2016). miRNA biogenesis: a dynamic pathway. Trends Plant Sci.

[2] Hammond SM (2015). An overview of microRNAs. Adv Drug Deliv Rev.

[3] Zhang L, Liao Y, Tang L (2019). MicroRNA-34 family: a potential tumor suppressor and therapeutic candidate in cancer. J Exp Clin Cancer Res.

[4] Otto T, Candido SV, Pilarz MS, Sicinska E, Bronson RT, Bowden M, Lachowicz IA, Mulry K, Fassl A, Han RC (2017). Cell cycle-targeting microRNAs promote differentiation by enforcing cell-cycle exit. Proc Natl Acad Sci USA.

[5] Bartel DP (2004). MicroRNAs: genomics, biogenesis, mechanism, and function. Cell.

[6] Stavast CJ, Erkeland SJ (2019). The Non-Canonical Aspects of MicroRNAs: Many Roads to Gene Regulation. Cells.

[7] Hermeking H (2010). The miR-34 family in cancer and apoptosis. Cell Death Differ.

[8] Nan L, Hongli Y, Liangui Y (2021). Exploring the influence of microRNA miR-34 on p53 dynamics: a numerical study project supported by the national natural science foundation of china under grant No. 11762011. Commun Theor Phys.

[9] Wang Y, Wang X, Wang M, Zhang L, Zan L, Yang W (2021). Bta-miR-34b controls milk fat biosynthesis via the Akt/mTOR signaling pathway by targeting RAI14 in bovine mammary epithelial cells. J Anim Sci Biotechnol.

[10] Jauhari A, Yadav S (2019). MiR-34 and MiR-200: regulator of cell fate plasticity and neural development. Neuromolecular Med.

[11] Misso G, Di Martino MT, De Rosa G, Farooqi AA, Lombardi A, Campani V, Zarone MR, Gullà A, Tagliaferri P, Tassone P (2014). Mir-34: a new weapon against cancer?. Mol Ther Nucleic Acids.

[12] Agostini M, Knight RA (2014). miR-34: from bench to bedside. Oncotarget.

[13] Wang R, Ma J, Wu Q, Xia J, Miele L, Sarkar FH, Wang Z (2013). Functional role of miR-34 family in human cancer. Curr Drug Targets.

[14] Zhan Y, Han J, Xia J, Wang X (2021). Berberine Suppresses mice depression behaviors and promotes hippocampal neurons growth through regulating the miR-34b-5p/miR-470-5p/BDNF Axis. Neuropsychiatr Dis Treat.

[15] Qi D, Hou X, Jin C, Chen X, Pan C, Fu H, Song L, Xue J (2021). HNSC exosome-derived MIAT improves cognitive disorders in rats with vascular dementia via the miR-34b-5p/CALB1 axis. Am J Transl Res.

[16] Tian J, Cui P, Li Y, Yao X, Wu X, Wang Z, Li C (2020). LINC02418 promotes colon cancer progression by suppressing apoptosis via interaction with miR-34b-5p/BCL2 axis. Cancer Cell Int.

[17] Guo X, Zhou Q, Su D, Luo Y, Fu Z, Huang L, Li Z, Jiang D, Kong Y, Li Z (2020). Circular RNA circBFAR promotes the progression of pancreatic ductal adenocarcinoma via the miR-34b-5p/MET/Akt axis. Mol Cancer.

[18] Dong L, Chen F, Fan Y, Long J (2020). MiR-34b-5p inhibits cell proliferation, migration and invasion through targeting ARHGAP1 in breast cancer. American J Transl Res.

[19] Zeljic K, Jovanovic I, Jovanovic J, Magic Z, Stankovic A, Supic G (2018). MicroRNA meta-signature of oral cancer: evidence from a meta-analysis. Ups J Med Sci.

[20] Inamoto T, Uehara H, Akao Y, Ibuki N, Komura K, Takahara K, Takai T, Uchimoto T, Saito K, Tanda N (2018). A panel of microRNA signature as a tool for predicting survival of patients with urothelial carcinoma of the bladder. Dis Markers.

[21] Zheng C, Wu D, Shi S, Wang L (2021). miR-34b-5p promotes renal cell inflammation and apoptosis by inhibiting aquaporin-2 in sepsis-induced acute kidney injury. Ren Fail.

[22] Wang H, Meng Q, Qian J, Li M, Gu C, Yang Y (2022). Review: RNA-based diagnostic markers discovery and therapeutic targets development in cancer. Pharmacol Ther.

[23] Barad O, Mann M, Chapnik E, Shenoy A, Blelloch R, Barkai N, Hornstein E (2012). Efficiency and specificity in microRNA biogenesis. Nat Struct Mol Biol.

[24] Zhao Y, Du T, Du L, Li P, Li J, Duan W, Wang Y, Wang C (2019). Long noncoding RNA LINC02418 regulates MELK expression by acting as a ceRNA and may serve as a diagnostic marker for colorectal cancer. Cell Death Dis.

[25] Liz J, Esteller M (2016). lncRNAs and microRNAs with a role in cancer development. Biochim Biophys Acta.

[26] Radha G, Raghavan SC (2017). BCL2: A promising cancer therapeutic target. Biochim Biophys Acta Rev Cancer.

[27] Chen S, Shen X (2020). Long noncoding RNAs: functions and mechanisms in colon cancer. Mol Cancer.

[28] Wang Y, Lin C, Liu Y (2021). Molecular mechanism of miR-34b-5p and RNA binding protein HuR binding to lncRNA OIP5-AS1 in colon cancer cells. Cancer Gene Ther.

[29] Kim J, Abdelmohsen K, Yang X, De S, Grammatikakis I, Noh JH, Gorospe M (2016). LncRNA OIP5-AS1/cyrano sponges RNA-binding protein HuR. Nucleic Acids Res.

[30] Jiang X, Ye Z, Jiang Y, Yu W, Fang Q (2020). LncRNA OIP5-AS1 upregulates snail expression by sponging miR-34a to promote ovarian carcinoma cell invasion and migration. Biol Res.

[31] Cesana M, Cacchiarelli D, Legnini I, Santini T, Sthandier O, Chinappi M, Tramontano A, Bozzoni I (2011). A long noncoding RNA controls muscle differentiation by functioning as a competing endogenous RNA. Cell.

[32] Luo G, Zhang Y, Wu Z, Zhang L, Liang C, Chen X (2021). Exosomal LINC00355 derived from cancer-associated fibroblasts promotes bladder cancer cell resistance to cisplatin by regulating miR-34b-5p/ABCB1 axis. Acta Biochim Biophys Sin.

[33] Nosol K, Romane K, Irobalieva RN, Alam A, Kowal J, Fujita N, Locher KP (2020). Cryo-EM structures reveal distinct mechanisms of inhibition of the human multidrug transporter ABCB1. Proc Natl Acad Sci USA.

[34] Fu W, Hong Z, You X, Din J, Chen B, Zhao B, Yuan G, Li Q (2019). Enhancement of anticancer activity of docetaxel by combination with Fuzheng Yiliu decoction in a mouse model of castration-resistant prostate cancer. Biomed Pharmacother.

[35] Yang C, Lu W, He H, Liu H (2020). Inflammation and DNA methylation-dependent down-regulation of miR-34b-5p mediates c-MYC expression and CRL4(DCAF4) E3 ligase activity in colitis-associated cancer. Am J Pathol.

[36] Halappanavar S, Wu D, Williams A, Kuo B, Godschalk RW, Van Schooten FJ, Yauk CL (2011). Pulmonary gene and microRNA expression changes in mice exposed to benzo(a)pyrene by oral gavage. Toxicology.

[37] Shi Z, Dragin N, Miller ML, Stringer KF, Johansson E, Chen J, Uno S, Gonzalez FJ, Rubio CA, Nebert DW (2010). Oral benzo[a]pyrene-induced cancer: two distinct types in different target organs depend on the mouse Cyp1 genotype. Int J Cancer.

[38] Tanaka N, Toyooka S, Soh J, Kubo T, Yamamoto H, Maki Y, Muraoka T, Shien K, Furukawa M, Ueno T (2012). Frequent methylation and oncogenic role of microRNA-34b/c in small-cell lung cancer. Lung Cancer.

[39] Wang Z, Chen Z, Gao Y, Li N, Li B, Tan F, Tan X, Lu N, Sun Y, Sun J (2011). DNA hypermethylation of microRNA-34b/c has prognostic value for stage I non-small cell lung cancer. Cancer Biol Ther.

[40] Landi MT, Zhao Y, Rotunno M, Koshiol J, Liu H, Bergen AW, Rubagotti M, Goldstein AM, Linnoila I, Marincola FM (2010). MicroRNA expression differentiates histology and predicts survival of lung cancer. Clin Cancer Res.

[41] May CD, Landers SM, Bolshakov S, Ma X, Ingram DR, Kivlin CM, Watson KL, Sannaa GAA, Bhalla AD, Wang WL (2017). Co-targeting PI3K, mTOR, and IGF1R with small molecule inhibitors for treating undifferentiated pleomorphic sarcoma. Cancer Biol Ther.

[42] 张艺, 尚亚峰, 孙建涛, 李小辉, 魏澎涛: 上调miR-34b-5p通过抑制胰岛素样生长因子1受体干扰肾癌Caki-1细胞的增殖和侵袭. 临床与实验病理学杂志 2019, 35(06):664–669.

[43] Butz H, Nofech-Mozes R, Ding Q, Khella HWZ, Szabó PM, Jewett M, Finelli A, Lee J, Ordon M, Stewart R (2016). Exosomal microRNAs are diagnostic biomarkers and can mediate cell-cell communication in renal cell carcinoma. Eur Urol Focus.

[44] Juan D, Alexe G, Antes T, Liu H, Madabhushi A, Delisi C, Ganesan S, Bhanot G, Liou LS (2010). Identification of a microRNA panel for clear-cell kidney cancer. Urology.

[45] Maroof H, Islam F, Ariana A, Gopalan V, Lam AK (2017). The roles of microRNA-34b-5p in angiogenesis of thyroid carcinoma. Endocrine.

[46] Maroof H, Islam F, Dong L, Ajjikuttira P, Gopalan V, Mcillan NAJ, Lam AK (2018). Liposomal delivery of miR-34b-5p induced cancer cell death in thyroid carcinoma. Cells.

[47] Plummer PN, Freeman R, Taft RJ, Vider J, Sax M, Umer BA, Gao D, Johns C, Mattick JS, Wilton SD (2013). MicroRNAs regulate tumor angiogenesis modulated by endothelial progenitor cells. Cancer Res.

[48] Price TJ, Tang M, Gibbs P, Haller DG, Peeters M, Arnold D, Segelov E, Roy A, Tebbutt N, Pavlakis N (2018). Targeted therapy for metastatic colorectal cancer. Expert Rev Anticancer Ther.

[49] Frezzetti D, Gallo M, Maiello MR, D'Alessio A, Esposito C, Chicchinelli N, Normanno N, De Luca A (2017). VEGF as a potential target in lung cancer. Expert Opin Ther Targets.

[50] Deng L, Jiang L, Tseng KF, Liu Y, Zhang X, Dong R, Lu Z, Wang X (2018). Aberrant NEAT1_1 expression may be a predictive marker of poor prognosis in diffuse large B cell lymphoma. Cancer Biomark.

[51] Hydbring P, Malumbres M, Sicinski P (2016). Non-canonical functions of cell cycle cyclins and cyclin-dependent kinases. Nat Rev Mol Cell Biol.

[52] Qian CS, Li LJ, Huang HW, Yang HF, Wu DP (2020). MYC-regulated lncRNA NEAT1 promotes B cell proliferation and lymphomagenesis via the miR-34b-5p-GLI1 pathway in diffuse large B-cell lymphoma. Cancer Cell Int.

[53] Tomasetti M, Amati M, Santarelli L, Neuzil J (2016). MicroRNA in metabolic re-programming and their role in tumorigenesis. Int J Mol Sci.

[54] Li Z, Luo Q, Xu H, Zheng M, Abdalla BA, Feng M, Cai B, Zhang X, Nie Q, Zhang X (2017). MiR-34b-5p suppresses melanoma differentiation-associated gene 5 (MDA5) signaling pathway to promote avian leukosis virus subgroup J (ALV-J)-infected cells proliferaction and ALV-J Replication. Front Cell Infect Microbiol.

[55] Lee CC, Wu CC, Lin TL (2014). Chicken melanoma differentiation-associated gene 5 (MDA5) recognizes infectious bursal disease virus infection and triggers MDA5-related innate immunity. Arch Virol.

[56] Lv H, Liu Q, Wen Z, Feng H, Deng X, Ci X (2017). Xanthohumol ameliorates lipopolysaccharide (LPS)-induced acute lung injury via induction of AMPK/GSK3β-Nrf2 signal axis. Redox Biol.

[57] Xie W, Lu Q, Wang K, Lu J, Gu X, Zhu D, Liu F, Guo Z (2018). miR-34b-5p inhibition attenuates lung inflammation and apoptosis in an LPS-induced acute lung injury mouse model by targeting progranulin. J Cell Physiol.

[58] Jiang K, Yang J, Guo S, Zhao G, Wu H, Deng G (2019). Peripheral circulating exosome-mediated delivery of miR-155 as a novel mechanism for acute lung inflammation. Mol Ther.

[59] Qiu N, Xu X, He Y (2020). LncRNA TUG1 alleviates sepsis-induced acute lung injury by targeting miR-34b-5p/GAB1. BMC Pulm Med.

[60] 顾晓丽, 陈芳: MiR-34b-5p通过靶向PGRN减轻LPS诱导的急性呼吸窘迫综合征大鼠的肺部细胞凋亡. *热带医学杂志* 2021, 21(06):705–710+817.

[61] Du X, Yang Y, Xiao G, Yang M, Yuan L, Qin L, He R, Wang L, Wu M, Wu S (2020). Respiratory syncytial virus infection-induced mucus secretion by down-regulation of miR-34b/c-5p expression in airway epithelial cells. J Cell Mol Med.

[62] Zanin M, Baviskar P, Webster R, Webby R (2016). The interaction between respiratory pathogens and mucus. Cell Host Microbe.

[63] Sigurs N, Aljassim F, Kjellman B, Robinson PD, Sigurbergsson F, Bjarnason R, Gustafsson PM (2010). Asthma and allergy patterns over 18 years after severe RSV bronchiolitis in the first year of life. Thorax.

[64] Peng F, He J, Loo JF, Yao J, Shi L, Liu C, Zhao C, Xie W, Shao Y, Kong SK (2016). Identification of microRNAs in throat swab as the biomarkers for diagnosis of influenza. Int J Med Sci.

[65] Giannandrea M, Parks WC (2014). Diverse functions of matrix metalloproteinases during fibrosis. Dis Model Mech.

[66] Leco KJ, Waterhouse P, Sanchez OH, Gowing KL, Poole AR, Wakeham A, Mak TW, Khokha R (2001). Spontaneous air space enlargement in the lungs of mice lacking tissue inhibitor of metalloproteinases-3 (TIMP-3). J Clin Invest.

[67] Hu RP, Lu YY, Zhang XJ (2019). MiR-34b-5p knockdown attenuates bleomycin-induced pulmonary fibrosis by targeting tissue inhibitor of metalloproteinase 3 (TIMP3). Eur Rev Med Pharmacol Sci.

[68] Li Y, Zhuo ZJ, Zhou H, Liu J, Xiao Z, Xiao Y, He J, Liu Z (2019). miR-34b/c rs4938723 T>C decreases neuroblastoma risk: a replication study in the hunan children. Dis Markers.

[69] Zeng Y, Li N, Zheng Z, Chen R, Peng M, Liu W, Zhu J, Zeng M, Cheng J, Hong C (2021). Screening of hub genes associated with pulmonary arterial hypertension by integrated bioinformatic analysis. Biomed Res Int.

[70] Hao S, Jiang P, Xie L, Xiang G, Liu Z, Hu W, Wu Q, Jiang L, Xiao Y, Li S (2021). Essential Genes and MiRNA-mRNA Network Contributing to the Pathogenesis of Idiopathic Pulmonary Arterial Hypertension. Front Cardiovasc Med.

[71] Li C, Zhang Z, Xu Q, Shi R (2020). Comprehensive analyses of miRNA-mRNA network and potential drugs in idiopathic pulmonary arterial hypertension. Biomed Res Int.

[72] Wang W, Chen J, Luo L, Li Y, Liu J, Zhang W (2018). Effect of cadmium on kitl pre-mRNA alternative splicing in murine ovarian granulosa cells and its associated regulation by miRNAs. J Appl Toxicol.

[73] 陈洁: mmu-miR-27a-3p、mmu-miR-34b-5p、mmu-miR-297a-3p、mmu-miR-129–5p、mmu-miR-107–3p在镉对小鼠卵巢颗粒细胞Kitl pre-mRNA选择性剪接影响中的变化. 硕士. 福建医科大学; 2016.

[74] Baker BC, Mackie FL, Lean SC, Greenwood SL, Heazell AEP, Forbes K, Jones RL (2017). Placental dysfunction is associated with altered microRNA expression in pregnant women with low folate status. Mol Nutr Food Res.

[75] Ali A, Hadlich F, Abbas MW, Iqbal MA, Tesfaye D, Bouma GJ, Winger QA, Ponsuksili S (2021). MicroRNA-mRNA networks in pregnancy complications: a comprehensive downstream analysis of potential biomarkers. Int J Mol Sci.

[76] Sree S, Radhakrishnan K, Indu S, Kumar PG (2014). Dramatic changes in 67 miRNAs during initiation of first wave of spermatogenesis in Mus musculus testis: global regulatory insights generated by miRNA-mRNA network analysis. Biol Reprod.

[77] Smorag L, Zheng Y, Nolte J, Zechner U, Engel W, Pantakani DVK (2012). MicroRNA signature in various cell types of mouse spermatogenesis: evidence for stage-specifically expressed miRNA-221, -203 and -34b-5p mediated spermatogenesis regulation. Biol Cell.

[78] Zhang HT, Zhang Z, Hong K, Tang WH, Liu DF, Mao JM, Yang YZ, Lin HC, Jiang H (2020). Altered microRNA profiles of testicular biopsies from patients with nonobstructive azoospermia. Asian J Androl.

[79] Eikmans M, Jacqueline DHA, Blijleven L, Meuleman T, van Beelen E, van der Hoorn MP, Claas FHJ (2020). Optimization of microRNA acquirement from seminal plasma and identification of diminished seminal microRNA-34b as indicator of low semen concentration. Int J Mol Sci.

[80] Sakurai K, Mikamoto K, Shirai M, Iguchi T, Ito K, Takasaki W, Mori K (2015). MicroRNA profiling in ethylene glycol monomethyl ether-induced monkey testicular toxicity model. J Toxicol Sci.

[81] Sakurai K, Mikamoto K, Shirai M, Iguchi T, Ito K, Takasaki W, Mori K (2016). MicroRNA profiles in a monkey testicular injury model induced by testicular hyperthermia. J Appl Toxicol.

[82] Zhou Y, Tian W, Zhang M, Ren T, Sun G, Jiang R, Han R, Kang X, Yan F (2019). Transcriptom analysis revealed regulation of dexamethasone induced microRNAs in chicken thymus. J Cell Biochem.

[83] Avgan N, Sutherland HG, Spriggens LK, Yu C, Ibrahim O, Bellis C, Haupt LM, Shum DH, Griffiths LR (2017). BDNF variants may modulate long-term visual memory performance in a healthy cohort. Int J Mol Sci.

[84] Ge QD, Xie C, Zhang H, Tan Y, Wan CW, Wang WJ, Jin TX (2019). Differential expression of miRNAs in the hippocampi of offspring rats exposed to fluorine combined with aluminum during the embryonic stage and into adulthood. Biol Trace Elem Res.

[85] Liu L, Liu L, Shi J, Tan M, Xiong J, Li X, Hu Q, Yi Z, Mao D (2016). MicroRNA-34b mediates hippocampal astrocyte apoptosis in a rat model of recurrent seizures. BMC Neurosci.

[86] Czabotar PE, Lessene G, Strasser A, Adams JM (2014). Control of apoptosis by the BCL-2 protein family: implications for physiology and therapy. Nat Rev Mol Cell Biol.

[87] Wang Q, Yu S, Simonyi A, Sun GY, Sun AY (2005). Kainic acid-mediated excitotoxicity as a model for neurodegeneration. Mol Neurobiol.

[88] Liu L, Liu L, Shi J, Tan M, Xiong J, Li X, Hu Q, Yi Z, Da M (2016). MicroRNA-34b mediates hippocampal astrocyte apoptosis in a rat model of recurrent seizures. BMC Neurosci.

[89] Rupaimoole R, Slack FJ (2017). MicroRNA therapeutics: towards a new era for the management of cancer and other diseases. Nat Rev Drug Discov.

[90] Li Y, Fang J, Zhou Z, Zhou Q, Sun S, Jin Z, Xi Z, Wei J (2020). Downregulation of lncRNA BACE1-AS improves dopamine-dependent oxidative stress in rats with Parkinson's disease by upregulating microRNA-34b-5p and downregulating BACE1. Cell Cycle.

[91] Koelsch G (2017). BACE1 function and inhibition: implications of intervention in the amyloid pathway of Alzheimer’s disease pathology. Molecules.

[92] Kabaria S, Choi DC, Chaudhuri AD, Mouradian MM, Junn E (2015). Inhibition of miR-34b and miR-34c enhances α-synuclein expression in Parkinson's disease. FEBS Lett.

[93] Sun N, Lei L, Wang Y, Yang C, Liu Z, Li X, Zhang K (2016). Preliminary comparison of plasma notch-associated microRNA-34b and -34c levels in drug naive, first episode depressed patients and healthy controls. J Affect Disord.

[94] Hitoshi S, Seaberg RM, Koscik C, Alexson T, Kusunoki S, Kanazawa I, Tsuji S, van der Kooy D (2004). Primitive neural stem cells from the mammalian epiblast differentiate to definitive neural stem cells under the control of Notch signaling. Genes Dev.

[95] Mizutani K, Yoon K, Dang L, Tokunaga A, Gaiano N (2007). Differential Notch signalling distinguishes neural stem cells from intermediate progenitors. Nature.

[96] Smalheiser NR, Lugli G, Rizavi HS, Torvik VI, Turecki G, Dwivedi Y (2012). MicroRNA expression is down-regulated and reorganized in prefrontal cortex of depressed suicide subjects. PLoS ONE.

[97] Hsieh CH, Rau CS, Jeng JC, Chen YC, Lu TH, Wu CJ, Wu YC, Tzeng SL, Yang JC (2012). Whole blood-derived microRNA signatures in mice exposed to lipopolysaccharides. J Biomed Sci.

[98] Suh SH, Lee KE, Kim IJ, Kim O, Kim CS, Choi JS, Choi HI, Bae EH, Ma SK, Lee JU (2015). Alpha-lipoic acid attenuates lipopolysaccharide-induced kidney injury. Clin Exp Nephrol.

[99] Naylor EC, Watson RE, Sherratt MJ (2011). Molecular aspects of skin ageing. Maturitas.

[100] Li T, Yan X, Jiang M, Xiang L (2016). The comparison of microRNA profile of the dermis between the young and elderly. J Dermatol Sci.

[101] Christoffersen NR, Shalgi R, Frankel LB, Leucci E, Lees M, Klausen M, Pilpel Y, Nielsen FC, Oren M, Lund AH (2010). p53-independent upregulation of miR-34a during oncogene-induced senescence represses MYC. Cell Death Differ.

[102] Yamakuchi M, Lowenstein CJ (2009). MiR-34, SIRT1 and p53: the feedback loop. Cell Cycle.

[103] Rokavec M, Li H, Jiang L, Hermeking H (2014). The p53/miR-34 axis in development and disease. J Mol Cell Biol.

[104] Li H, Wang X, Lu X, Zhu H, Li S, Duan S, Zhao X, Zhang F, Alterovitz G, Wang F (2019). Co-expression network analysis identified hub genes critical to triglyceride and free fatty acid metabolism as key regulators of age-related vascular dysfunction in mice. Aging.

[105] Victoria B, Dhahbi JM, Nunez Lopez YO, Spinel L, Atamna H, Spindler SR, Masternak MM (2015). Circulating microRNA signature of genotype-by-age interactions in the long-lived Ames dwarf mouse. Aging Cell.

